# Development and testing of the IMPRESS hospital management survey (IHMS) to measure hospital management practices in a low-income country setting

**DOI:** 10.1186/s12913-025-13704-7

**Published:** 2025-11-20

**Authors:** Charlotte Ward, Wanangwa Chimwaza, Monica Malata, Elias Rejoice Maynard Phiri, Alilane Linda Nyondo-Mipando, Andrew Kumitawa, Vincent Samuel Phiri, Catherine Goodman, Victor Mwapasa, Timothy Powell-Jackson

**Affiliations:** 1 London School of Hygiene and Tropical Medicine, 15-17 Tavistock Pl, London, WC1H 9SH UK; 2 Kamuzu University of Health Sciences, Blantyre, Malawi

**Keywords:** Management practices, Tool, Reliability, Validity, Acceptability, Measurement, Malawi, Hospitals

## Abstract

**Background:**

Strong hospital management has long been recognised by policymakers and the public as important for hospital performance, yet it has received limited empirical research attention. This paper aims to describe the rigorous development and performance testing of a tool to measure hospital management practices in Malawian hospitals.

**Methods:**

Using a systematic process informed by a scoping review of existing tools, government policies and guidelines, qualitative interviews with hospital managers, a team workshop, and pilot testing, we developed a tool to assess 28 management practices across five domains: clinical care delivery in the neonatal unit, human resource management, target setting and monitoring, financial management, and leadership and governance. The tool was implemented by trained data collectors in 36 Malawian hospitals between April and May 2022, with five managers interviewed per hospital (*n* = 180). Guided by psychometric techniques and supplemented by qualitative feedback from 36 hospital dissemination meetings, we evaluated the tool’s acceptability, reliability and validity, and compare the primary measure of management with two alternative scoring methods.

**Findings:**

Our findings are threefold: first, the tool was feasible to implement and acceptable to the interviewees. Second, it demonstrated strong psychometric performance: factor analysis showed that one principal component loaded positively on all the management practices, thus the tool measures a single latent factor of hospital management. Results from item analysis indicate that the tool detected variation between the hospitals for each management practice and none of the management practices are redundant, with low inter-item correlation. Finally, the primary management measure was highly correlated with alternative scoring methods.

**Interpretation:**

To our knowledge, this is the first study to describe the systematic development and rigorous validation of a tool that quantitatively measures management practices in a low-resource, hospital setting. We discuss methodological insights and suggest adaptations for implementing the tool in other low-income settings. This paper contributes to the limited evidence base on the development and performance testing of tools for quantitatively measuring management practices in the healthcare sector.

**Supplementary Information:**

The online version contains supplementary material available at 10.1186/s12913-025-13704-7.

## Introduction

Strong hospital management has long been recognised by policymakers and the public as important for hospital performance, yet it has received limited empirical research attention [1, 2]. This is particularly evident in low- and middle-income countries where the prevailing focus has been on the effectiveness of health technologies [3], medical training and clinical audits [3] on quality of care, with less research attention on organisation-level factors that shape health service delivery [4].

Empirical research on organisations is important. Key research questions, such as which aspects of management, organisational culture, or leadership impact hospital performance and how these can be improved, have long been on the agenda [2]. To explore these issues, qualitative approaches often employ case studies, which create a rich picture of how management practices work and under what conditions [5, 6]. These approaches can be particularly valuable for generating hypotheses. Quantitative methods may complement these approaches by systematically collecting data across a large number of hospitals to identify patterns and understand variation at scale. However, while well-established metrics exist for assessing hospital performance, measuring organisational behaviour is more challenging. Firstly, defining organisational concepts such as “leadership” and “management” is complicated because they are multi-faceted and the boundaries around each term overlap to some degree. Secondly, it not always easy to determine whether a particular management practice is beneficial or detrimental to organisational performance. For example, conducting hospital staff appraisals is generally considered to be a “good” practice, but its impact on hospital performance may depend on the quality of the appraisal discussion. Third, management practices are often difficult to observe and, therefore, challenging to measure. One way to address this is by assessing evidence of adherence to these practices, such as reviewing management-related tools or documents like meeting minutes. However, the extent to which this approach measures the quality of the management practice remains debatable. Lastly, when attempting to measure a complex and multi-faceted construct, feasibility of administering measurement tools becomes an issue.

There has been substantial progress in addressing these challenges. A pioneering area is the World Management Survey (WMS), which researchers have used to develop a global body of evidence on quantitative measurement of management practices [7, 8]. However, the WMS was designed to measure management in a comparable way across different sectors and industries, not health per se. Moreover, it was originally developed for high-income settings, which has implications for both the tool and the approach to data collection. For instance, certain basic organisational functions, such as financial management, are not addressed in the survey, even though they are critical barriers to progress in other contexts. In addition to WMS, there is also a small evidence base describing the development and performance testing of management survey tools in primary health care settings [9, 10], at the district level [11] and among commissioners in the English National Health Service (NHS) [12]. However, the current evidence base lacks research on appropriate tools for measuring management practices in hospitals in a low-income setting.

We systematically develop a tool to measure hospital management practices in Malawi as part of the wider IMPRESS project (Innovative Management PRactices to Enhance hoSpital quality and Save lives in Malawi). IMPRESS examines whether improving hospital management can enhance the quality of care for small and sick neonates. We theorise that adoption of structured management practices – widely recognised as integral to a well performing organisation –can improve the way health workers deliver care to small and sick newborns, for example by enhancing clinical skills and knowledge, staff motivation, teamwork and communication, ultimately leading to improved clinical quality of care in the neonatal unit. Measuring the strength of management practices is the first step in enabling hospitals to understand their performance and make the necessary changes to improve management.

Building on existing tools and best practice, this study addresses three objectives. First, we describe the systematic, evidence-based development of the IMPRESS Hospital Management Survey (IHMS) tool. Second, using data collected from 36 hospitals in Malawi, we assess the tool’s acceptability, reliability, and validity. Third, we compare the IHMS score with two simpler alternatives.

## Methods

In this section, we describe the process to develop the IHMS tool, and the data collection procedures and statistical analyses used to evaluate it. All these methods speak to the acceptability, reliability, and validity of the tool, and provide a template for how other researchers can adapt the tool to different contexts.

### Conceptualisation of management

Defining hospital management poses practical challenges due to its broad and multi-disciplinary conceptualisation, which spans various bodies of literature. There is therefore no single definition that is widely accepted. One common definition describes healthcare management as “continuously developing the potential of an organisation to transform human and financial resources and other inputs into improved services and better health” [13]. Others have defined management as “the control, monitoring or organisation of people, processes and systems in order to achieve specific goals” [14]. These definitions, while a useful starting point, are broad and only take us so far. For our purposes the literature is arguably more useful when it conceptualises management according to various domains. For example, the WMS describes four domains of management: operations management, talent management, performance management and targets [15–17] and has formed the basis of many subsequent tools.

The literature identifies several distinctions that help conceptualise hospital management. One distinction is between management and leadership. While these roles are closely interconnected and often held by the same individual, they differ in focus, with leadership involving influencing and inspiring others to achieve common goals [18]. A related body of research examines the role of clinicians as hospital leaders and their impact on hospital performance outcomes [19]. Another important distinction is between the quantity and quality of management. In contexts like the UK’s NHS, public discourse often focuses on the quantity of management, with concerns about excessive bureaucracy and the number of managers. Research in this area explores how the number of managers relates to hospital performance [20]. Additionally, there is a distinction between managers and management processes. The former concerns the traits and qualities of what makes a good manager, while the latter focuses on the systems and practices designed to enhance organisational performance. Finally, there is a broader literature on the relationship between organisational culture and performance [21, 22]. This is closely linked to management, as the culture within which a manager operates can significantly influence their ability to drive change within the organization.

A complementary body of literature evaluates management interventions in healthcare settings, offering additional perspectives on how management can be conceptualised. Some studies focus on interventions aimed at enhancing the skills and competencies of managers [23–25], while others focus on evaluating the introduction of a new management system [26] or a performance-based accreditation system [27].

Understanding these conceptualisations of management and key distinctions helped us to define the scope of the IHMS. Our tool focuses on assessing organisational-level management practices, by which we mean actions taken to adhere to systems and processes across different functions of the hospital. We are interested in the quality with which these actions are taken, rather than the amount of time spent performing management practices and they can be performed by anyone, regardless of whether they have a managerial or leadership position. Such practices are distinct from clinical management, which involves actions taken by healthcare providers in delivering patient care. Drawing from WMS, we arrange the IHMS into domains of management and whilst our tool does not attempt to measure strength of leadership, we do include a leadership and governance domain which measures management practices related to leadership and governance functions in the hospital.

### Developing management domains and scoring practices

Between September 2021 and March 2022, we developed the IHMS tool using a systematic, evidence-based approach guided by best practices [28]. Firstly, a scoping review was conducted to identify existing tools to measure adoption of management practices in hospitals. This search formed a subset of a systematic literature review summarising evidence on the relationship between management and quality of care [29]. The authors reviewed literature published on the PubMed database from January 1st 2000 to March 2022. The search combined terms related to (1) providers of medical or health-related services at hospital level; (2) management measurement tools; (3) quality of care. The search produced an extensive list of management domains and practices that have been used to measure management across different income-settings (Supplementary File, Appendix 1). It also outlined key methodological decisions in measuring management, which guided the development of the IHMS tool.

Secondly, Government of Malawi policies and guidelines were reviewed to gather information on formal management systems and processes concerning hospitals. These included procedures and systems covering the whole of government, those specific to the health sector or hospitals, and specific clinical protocols for the care of infants and newborns, such as the Care of the Infant and Newborn in Malawi (COIN) guidelines. Third, the study conducted data collection across four hospitals: four in-depth interviews with hospital managers and three group model building exercises with frontline health workers. These activities aimed to understand the management-related challenges affecting the quality of care in Malawian hospitals. Fourthly, results from these activities were discussed at a five-day workshop between the Kamuzu University of Health Sciences (KUHeS) and London School of Hygiene and Tropical Medicine (LSHTM) team members, comprising a range of academic and professional backgrounds including implementation science, social science, health economics, clinical, nursing, statistics and data management. Participants identified the most relevant management domains and practices in Malawi and discussed what would be considered the best and worst case descriptors for each management practice. These final three steps were critical to tailoring the IHMS to the context in Malawi.

**Fig. 1:**
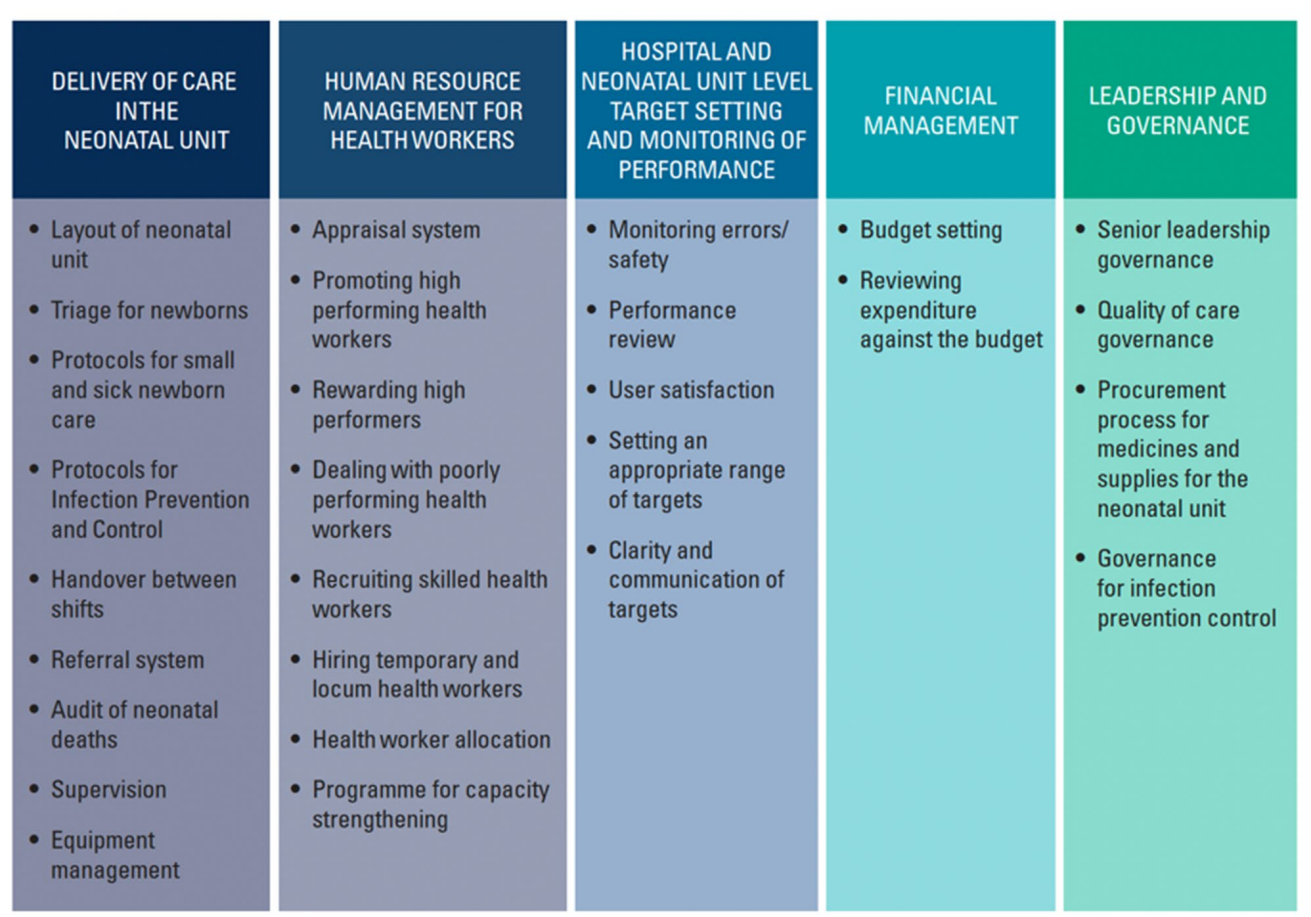
IHMS tool domains and practices

The resulting IHMS tool collects data about the characteristics of the hospital and the respondents. It then goes on to ask questions about adoption of 28 management practices across five domains: (i) delivery of care in the neonatal unit (9 practices), (ii) human resource management for health workers (8 practices), (iii) hospital and neonatal ward level target setting and monitoring of performance (5 practices), (iv) financial management (2 practices), and (v) leadership and governance (4 practices) (Fig. 1). It is important to emphasise that the domain ‘delivery of care in the neonatal unit’ examines 9 management practices that support care delivery, rather than assessing the actual delivery of care itself. Following the WMS method [7], the interviewee is asked several open-ended questions for each management practice, such as *‘Can you tell me about the referral system for receiving neonatal patients?’* and *‘How does the hospital communicate with the referring facility?’*. Open-ended questions allow the interviewer to engage in a conversation without imposing any strong direction. To improve objectivity in scoring, exact descriptors for each management practice for scores one (lowest), three and five (highest), were developed through extensive consultation. This process provided clear guidance to the interviewer for evaluating each management practice. For example, a score of one means *‘There is no system in place to standardise inward referral of patients; there is little communication between the referring facility and the hospital. There is no organised transport.’* A score of five means *‘A standardised process for receiving inward referrals exists. Referral communication is functional using a referral form and organised transport. There is feedback between facilities.’*

The IHMS also includes two alternative methods for collecting data on management practices. The first asks closed-ended questions about each of the 28 management practices and responses are recorded using ordered categorical response options, coded between 0 (worst) and 1 (best). The second is a record review which assesses 25 management items including meeting minutes, clinical manuals, administrative documentation, and forms (Supplementary File, Table 4). These are also scored on a 0 to 1 scale. For example, meeting minutes completed and dated within 3 months would receive a higher score than those dated more than 3 months ago. We collected these data to compare our main measure of management with alternative measures of management.

A half-day pilot of the IHMS with a Matron and District Medical Officer from a district hospital aimed to (a) test the tool content and language; (b) test the practicalities of the interview including the length of time taken to complete the interview and the roles of the interviewees; (c) allow interviewees to practice interview technique. There were minor changes to the tool’s language after the pilot to ensure that it was understood by target respondents. The finalised tool is publicly available for others to use and adapt [30].

### Data collection procedures

We hired 10 research assistants with a post-graduate public health or related qualification and experience working in Malawian hospitals to administer the IHMS. We trained them over a one-week period to ensure that they had a consistent understanding of the meaning of the questions and the terms used. The training included multiple mock interviews, group scoring exercises designed to calibrate management scoring across interviewers on the 1 to 5 scale, and skills in probing on actual practices and strategies being used in the hospital in order to elucidate direct personal experiences.

Between April and May 2022, the IHMS tool was administered in 36 Malawian hospitals using Open Data Kit for tablet-assisted data entry. The sample included all four central government hospitals, 24 district hospitals, and the eight largest Christian Health Association of Malawi (CHAM) hospitals. These hospitals capture the vast majority of secondary and tertiary care provision in the country. Hospitals excluded from the study were mental health or small rural government hospitals, small CHAM hospitals and the few private for-profit hospitals.

Research assistants conducted three-day visits to each study hospital. In this time, research assistants individually interviewed five managers using the IHMS and conducted one record review. Respondents were selected to represent varying levels of seniority and management responsibilities, both at the neonatal unit and hospital levels, including individuals with a broad overview of the organisation as well as hybrid clinician-managers with detailed knowledge of daily operations. Individuals answered different sections of the tool. This approach acknowledged that individuals in certain roles might be better informed about specific management practices. These roles were the Sister in-Charge of the neonatal unit, Unit Matron, Hospital Administrator, District Nursing Officer, and District Medical Officer.

Each interview was conducted by two research assistants. Respondents were not informed in advance that their responses would be scored quantitatively, and they did not observe the scoring process as research assistants completed the scoring discreetly during or after the interview. After administering each survey, the two research assistants discussed and reached consensus on the final scores for each practice. Where an individual score could not be agreed, further input was sought from a member of the core research team. Study investigators were also present in many of the interviews to supervise and review interviewer scores.

The IHMS tool also included three post-interview questions to be completed by the research assistants to record their perceptions of the respondents’ knowledge of management practices, willingness to reveal information, and patience in answering all the questions. These were scored on a scale of 1 (worst) to 5 (best) and give some indication of the perceived acceptability of the survey to the respondents.

After data analysis, dissemination meetings were held at each of the 36 hospitals in August 2022 with those who participated in the survey and other frontline health care staff. For each hospital, two research assistants presented the IHMS findings and facilitated a discussion to gather reactions to the hospital’s results, discuss domains they wanted to improve, strategies for improvement and how IMPRESS could provide support to the hospital in future. Meetings also provided an opportunity for hospitals to comment on the content of the survey. Written reports were compiled for each hospital and content summarised.

### Statistical analysis

To generate an overall score of management for a hospital, we proceed in two steps. First, for each of the 28 management practices, we took the mean across the respondents who were scored within each hospital. Second, we took the mean across the 28 management practices to produce an overall summary score of management that is a continuous measure. The implication of this method is that each management practice receives equal weight in the overall summary score. This aggregation method was used for both the open and closed-ended questions to generate two summary scores. The closed-ended score is a different method which we consider as a subset of the open-ended score, which is our primary measure of management i.e. the IHMS score. To generate the record review summary measure, we took the mean across the 26 items, which was interpreted as the proportion of the maximum score obtainable.

Guided by standard, commonly used psychometric techniques [31], we evaluated the acceptability, reliability and validity of the tool for providing scientifically credible information. Tests were performed at the item (management practice) level and on the IHMS score. Table 2 provides a summary of the tests performed, the acceptance criteria and the results of performance testing.

Firstly, we performed item analysis to identify management practices for possible elimination owing to weak performance. For management each practice, we analysed how frequently hospitals were scored in each response category (1 to 5) to determine whether any category was chosen in more than 80% of interviews and to identify practices that did not effectively differentiate between hospitals. Exploratory Factor Analysis (EFA) is an approach used to determine the underlying structures within data and identify whether questions are measuring one or more underlying construct. Principal Component Factor Analysis is a subset of EFA commonly used when the underlying structure of the data are not known. We evaluated all 28 management practices to determine their correlation with the first unrotated factor and assess whether each practice had a correlation above the desired threshold of 0.3. Inter-item correlation was used to identify redundancy of certain management practices exceeding a correlation threshold of 0.75.

We assessed the acceptability of the tool quantitatively by measuring the completeness of data, and floor and ceiling effects for the IHMS overall and domain-level score; and qualitatively, through discussions with respondents during the dissemination meetings. Perceived acceptability was measured form the perspective of the research assistant who answered three questions on the interviewee’s knowledge, willingness and patience. We also assessed the tool’s reliability, which is the degree to which the tool is free from measurement error [32], based on internal consistency and test-retest reliability. Here, we assessed for internal consistency of the IHMS score using Cronbach’s alpha to describe the extent to which management practices are closely related as a group, with a threshold of ≥ 0.70. We assessed an apparent test-retest reliability by measuring the within-hospital variation between management scores using the intra-class correlation coefficient. This was not strictly test-retest because we compare the scores between different managers within the same hospital. However, since we were trying to quantify each individual hospital’s management score, it remained useful to understand the extent to which managers had different views about management practices.

#### Correlation with other measures

To address methodological questions, we were interested in whether the score generated through the IHMS method is correlated with the two alternative methods – the closed-ended score and the record review score. We compared these scores using Pearson’s correlation coefficient.

## Results

### Descriptive statistics

Thirty-six hospitals participated in the management survey including district public (66.7%), central public (11.1%), and Christian Health Association of Malawi (CHAM) (22.2%) hospitals. A total of 180 managers were interviewed (five managers per hospital). The mean age of managers was 37 years (SD = 8.7) and 60% were female. Sixty-five (36.1%) managers had a qualification in management, with the most frequent being a certificate in management (18.3%) or a BSc in Health Management (6.1%).

Using the IHMS tool, the average overall IHMS score across all hospitals was 3.35 (SD = 0.41) (on a scale of 1 to 5). Domain level IHMS scores ranged from 2.87 (SD = 0.59) for “target setting and monitoring of performance”, to 3.63 (SD = 0.56) for “financial management” (Table 1). Across the 28 individual management practices, the average score was lowest for “promoting high-performing health workers” (mean = 2.23, SD = 0.63) and highest for “‘layout of the neonatal unit” (mean = 4.15, SD = 0.76) (Supplementary File, Table 5).

**Table 1:** IHMS score overall and by domain of management

	Mean	Std. deviation	Min	Max
**IHMS score overall**	3.35	0.41	2.44	4.24
**Domain level IHMS score (no. of practices)**				
Delivery of clinical care in the neonatal unit (9)	3.59	0.46	2.28	4.56
Human resource management for health workers (8)	3.18	0.53	2.15	4.28
Hospital and neonatal ward level target setting and monitoring of performance (5)	2.87	0.59	1.64	4.12
Financial management (2)	3.63	0.56	2.50	4.67
Leadership and governance (4)	3.58	0.53	1.95	4.40

### Item analysis

There were no missing data, and floor and ceiling effects were not present, with no hospital scoring the minimum score of 1 or the maximum score of 5 for the overall IHMS score or the domain level score (Table 1). None of the items had greater than 80% respondents across all hospitals endorsing the same response category. The maximum endorsement frequency was for the management practice “equipment” where 55.6% respondents were scored a ‘4’ on the 1 to 5 scale. In the inter-item correlation test, none of the 28 questions were redundant, with all items below the 0.75 threshold (Table 2; Supplementary File, Table 5), meaning that each item appeared to measure a distinct concept.

**Table 2:** Analytic tests of tool’s performance

Property	Analysis	Acceptance criteria	Result	Criterion met? (Y/*N*)	Interpretation
Item analysis	Item analysis	• Maximum endorsement frequency should be ≤ 80% (includes floor and ceiling effect ≤ 80%)	• No item failed	• Y	None of the items had > 80% respondents across hospitals endorsing the same response category.
		• Unrotated principal component factor analysis: o All items load positively onto the first factor o All items load on first unrotated factor > 0.3	• All items loaded positively onto the first factor • 2/28 failed: item 1 (layout) = 0.23 and item 5 (handover) = 0.26	• Y • N	The 28 items measure a single factor. 2/28 items are less correlated with the first factor.
		• Inter-item correlation ≤ 0.75	• All inter-item correlations ≤ 0.32	• Y	Low inter-item correlation suggests that none of the items are redundant.
Acceptability	Item analysis and data quality	• Floor and ceiling effect of mean summary score < 10%	• % floor: 0% hospitals scored 1 • % ceiling: 0% hospitals scored 5	• Y • Y	No hospitals obtained a minimum or maximum mean summary score.
		• Missing data < 5%	• % missing: 0%	• Y	Every hospital had a fully completed survey with no missing data.
Reliability	Internal consistency	• Cronbach’s alpha for summary score ≥ 0.70	• Cronbach’s alpha = 0.92	• Y	Management practices are closely related as a group.
	Test-retest	• Intraclass correlation (ICC)	• Domain 1*: ICC = 0.40 (95% CI 0.13, 0.68) • Domain 2: ICC = 0.39 (95% CI 0.23, 0.55) • Domain 3: ICC = 0.30 (95% CI 0.15, 0.48) • Domain 4: ICC = 0.13 (95% CI 0.00, 0.34) • Domain 5: ICC = 0.32 (95% CI 0.16, 0.48)	n/a	Evidence of low test-retest reliability between respondents for domain 4.

*Domain 1 = Delivery of care in the neonatal unit; Domain 2 = Human resource management for health workers; Domain 3 = Hospital and neonatal ward level target setting and monitoring of performance; Domain 4 = Financial management; Domain 5 = Leadership and governance

**Fig. 2:**
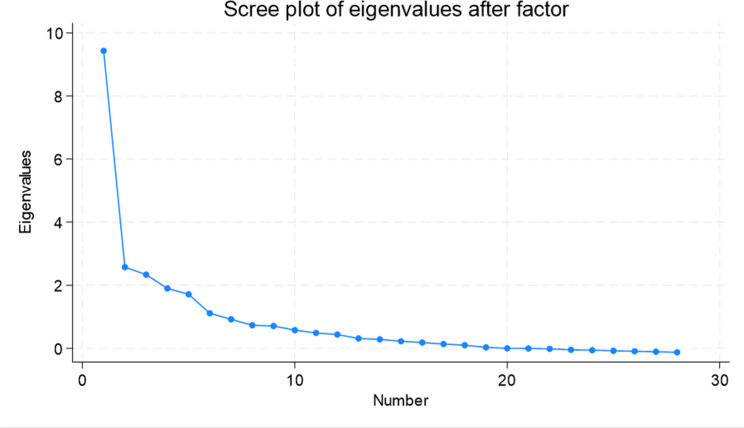
Scree plot from Principal Component Factor Analysis

The principal component factor analysis shows that all management practices load positively onto the first factor (Table 3). Item 1 (layout) and item 5 (handover) failed to load > 0.3 which suggests that these management practices are least correlated with the overall measure of management compared to the other 26 items. The scree plot, with a clear “elbow” after the first factor suggests that the variables in the analysis are highly correlated and can be largely explained by a single underlying construct, i.e. “good management” (Fig. 2).

**Table 3:** Results from the principal component factor analysis

No.	Management practice	Factor1	Factor2	Factor3	Factor4	Factor5	Factor6	Factor7	Uniqueness
1	Layout	0.23	0.14	0.42	0.34	0.46	-0.31	0.31	0.23
2	Triage	0.50	0.22	0.06	0.51	0.53	-0.04	-0.01	0.16
3	COIN protocols	0.61	0.05	0.31	0.37	0.26	0.21	0.01	0.28
4	IPC protocols	0.56	-0.27	0.05	0.49	-0.19	-0.09	-0.30	0.25
5	Handover	0.26	0.33	0.48	0.10	-0.27	0.38	-0.13	0.34
6	Referrals	0.51	0.15	0.23	0.16	-0.04	0.42	-0.52	0.19
7	Audit	0.54	0.23	-0.36	0.45	-0.28	0.03	0.15	0.23
8	Supervision	0.58	-0.24	0.19	0.37	-0.25	0.03	0.07	0.36
9	Equipment	0.71	-0.15	-0.09	0.35	-0.20	0.01	0.21	0.26
10	Appraisal	0.68	0.35	-0.11	-0.16	-0.37	-0.11	0.13	0.21
11	Promotion	0.58	0.34	0.42	-0.34	-0.09	-0.17	0.16	0.20
12	Reward	0.47	0.21	0.14	-0.25	-0.31	-0.53	-0.23	0.23
13	Poor performance	0.52	0.40	-0.09	-0.16	-0.36	0.26	0.26	0.27
14	Recruitment	0.71	0.42	0.19	-0.28	0.00	0.03	0.00	0.21
15	Temporary staff	0.71	0.34	-0.16	-0.01	-0.04	-0.18	-0.24	0.27
16	Staff allocation	0.74	0.06	-0.28	0.13	0.09	-0.32	-0.11	0.23
17	Capacity strengthening	0.68	-0.09	0.03	0.15	-0.04	-0.18	0.32	0.37
18	Monitoring errors	0.57	-0.20	0.29	-0.23	0.41	0.12	0.00	0.32
19	Performance review	0.72	-0.28	0.12	-0.29	0.11	0.10	-0.04	0.28
20	User satisfaction	0.64	0.20	-0.32	0.01	0.10	-0.11	-0.12	0.41
21	Target range	0.69	-0.48	-0.11	-0.21	0.06	0.01	0.02	0.24
22	Target communication	0.78	-0.22	-0.05	-0.30	-0.01	-0.01	-0.19	0.20
23	Budget setting	0.40	0.22	-0.68	-0.07	0.42	0.04	-0.18	0.12
24	Budget expenditure	0.42	0.41	-0.32	-0.31	0.48	0.25	0.18	0.13
25	Senior leadership governance	0.47	-0.24	0.71	-0.25	0.14	-0.07	0.03	0.13
26	Quality of care governance	0.70	-0.45	-0.19	-0.07	-0.02	0.21	0.06	0.21
27	Drug procurement	0.58	-0.38	-0.28	-0.08	-0.17	0.28	0.29	0.25
28	IPC governance	0.33	-0.71	-0.12	-0.11	0.01	-0.19	-0.10	0.31

COIN: Care of the Infant and Newborn in Malawi

IPC: Infection Prevention and Control

Research assistants rated respondents’ knowledge of management practices (mean score 3.9), their willingness to reveal information (mean score 4.7) and their patience when giving responses (mean score 4.7).

### Qualitative feedback

Feedback during the hospital dissemination meetings about the results of the survey was generally positive, with participants consistently expressing appreciation for receiving research feedback—a practice they noted was rare in their experience. Some hospitals readily accepted the results while others needed their clarifications before fully accepting their scores. A common pattern was that staff initially questioned their scores but accepted them after the criteria for best practices were explained in detail.

Several hospitals were interested in comparing their scores with other hospitals of the same type (District, Central or CHAM). Hospital staff wanted to understand more about the scoring process, particularly in areas needing improvement. Multiple hospitals requested access to the full IHMS tool, particularly so that they could understand what constitutes best practice.

In terms of the IHMS tool content, few fundamental concerns were raised. The main exception was the perspective that certain practices were not fully under the control of hospital staff. In particular, while hospitals could influence some Human Resources decisions, the ultimate responsibility for recruiting staff and promoting high performers in government hospitals lay with local government and the Ministry of Health (Health Service Commission). Related to this, several hospitals remarked that appraisals are supposed to be used by the Ministry of Health as evidence when considering promotion, but in practice, appraisals are rarely used for this purpose. Many hospitals reported that appraisals had been discontinued because there was no meaningful response or action from central authorities.

Some feedback indicated that questions relating to financial management were challenging to answer due to lack of transparency during budget setting and knowledge gaps about financial processes among general staff interviews. Some participants suggested that these questions may have been easier to answer by a specialist, such as the hospital accountant or the Director of Finance in the District Council.

### Internal consistency and within hospital variation

The overall management score showed good internal consistency, which is a measure of how closely related the management practices are as a group. The Cronbach’s alpha for the overall score was 0.92 which is above the 0.7 threshold (Supplementary File, Table 5). The degree of agreement between managers within the same hospital, as measured by the intraclass correlation, was highest for the delivery of care in the neonatal unit domain (ICC = 0.40). Respondents agreed to some degree on their scores within the human resource (ICC = 0.39), targets (ICC = 0.30) and leadership (ICC = 0.32) domains. Agreement between the respondents within a hospital was low for the financial management domain (ICC = 0.13) (Table 2).

### Correlation with other measures

**Fig. 3:**
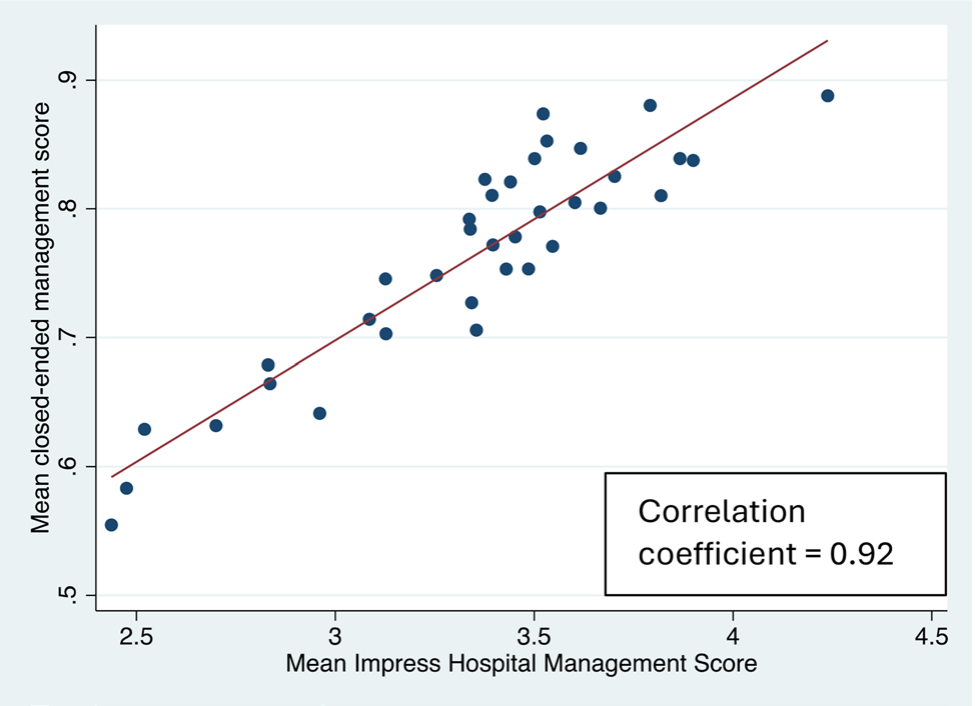
Scatter plot to show correlation between mean IMPRESS hospital management score and mean closed-ended management score

**Fig. 4:**
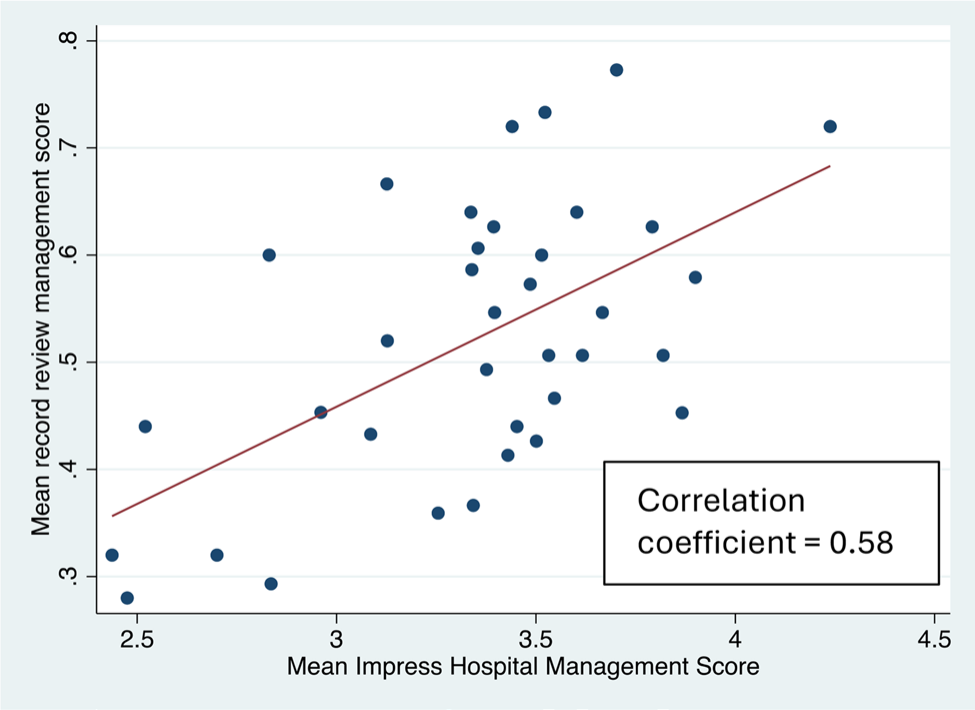
Scatter plot to show correlation between mean IMPRESS hospital management score and mean record review management score

When comparing the mean scores for the IHMS score and the closed-ended scores there is a high level of correlation (correlation coefficient = 0.92) (Fig. 3). The correlation between the record review and IHMS score was reasonable, with a correlation of 0.58 (Fig. 4).

## Discussion

In this paper, we describe the development of the IHMS tool to assess management practices in government and faith-based (CHAM) hospitals in Malawi. We then evaluate the tool’s acceptability, reliability and validity, and compare the primary IHMS score with two alternative methods.

To our knowledge, this is the first study to describe the systematic development and rigorous validation of a tool that quantitatively measures hospital management practices in a low-resource setting. Other studies have described the development and assessed the performance of tools measuring management practices in different settings including healthcare payors (commissioners) in England’s NHS [12], the district public health administration in India [11] and primary health care settings in Ghana [9] and Nigeria [10]. All tools were developed using an adapted World Management Survey approach and assessed management practices across several domains of management using in-person interviews. These tools demonstrated good psychometric performance across one or a range of measures.

The IHMS tool was feasible to implement and acceptable to the interviewees. It received positive feedback from hospitals, with respondents welcoming the opportunity to discuss these types of management issues in detail. It is worth noting that from a practical perspective, the collection of these data required considerable buy-in from the in-charge of the hospital and the hospital research committee, coupled with careful scheduling of interviews, to ensure that respondents would be able to take time away from their day to day duties for an interview lasting up to 90 min, during the three-day visit from the research team.

Results from the item analysis indicate good psychometric performance. As demonstrated by the maximum endorsement frequency testing, the tool detected variation between the hospitals for each management practice and no hospital received an overall IHMS score or domain level score of 1 (lowest) or 5 (highest). Almost all of the individual management practices were correlated with the overall management score and the factor analysis suggested that the tool is measuring a single factor. Qualitative feedback from the dissemination meetings indicated that the management practices measuring “promoting high performing health workers” and “recruiting skilled health workers on a permanent basis” had limited validity in measuring hospital management in government hospitals in Malawi because currently these human resource issues are managed by local government and the Ministry of Health (Health Service Commission).

The IHMS was administered to five different types of managers in the hospital, each with differing clinical and managerial responsibilities at the hospital and neonatal unit level. We assigned each manager to respond to specific sections of the tool based on their expertise, in contrast to the WMS method, where a single manager from each hospital completes the entire survey. An advantage of interviewing a range of respondents is that we could measure varied opinions regarding hospital-wide management systems and processes relating to human resources, finances, governance, and use of data, while also assessing day-to-day management practices within a specific hospital department - the neonatal unit. Interviewing up to five respondents per question can also help reduce measurement error by averaging the scores for each management practice across the respondents who answered that question.

By assessing intra-cluster correlation (ICC), we quantified the level of agreement among respondents. Our analysis revealed the lowest agreement in responses to financial management questions (ICC = 0.13) and the highest agreement in responses related to neonatal unit care delivery (ICC = 0.40). For context, a similar tool used in India’s public health system among district public health managers achieved a higher ICC of 0.52 [11]. Disagreement between managers in our study was somewhat expected, as we intentionally selected respondents with different management roles. The low agreement for the financial management domain suggests that these questions are less reliable. During our dissemination meeting, participants suggested that financial management questions would be more appropriately directed to hospital accountants or District Council Directors of Finance rather than general hospital managers. Future users of IHMS should consider whether this domain is suitable for selected respondents in their setting and if not, consider removing the domain.

Our primary measure of management – the IHMS score – was highly correlated with the score generated using closed-ended questions on the same 28 management practices. The closed-ended approach is quicker to administer than the IHMS approach and serves as a useful alternative with respect to the presence or absence of particular management practices. However, the IHMS approach has the advantage of the detailed 5-point scoring method, which is likely to be more sensitive to changes, either over time or cross-sectionally due to an intervention. It also offers the possibility, if the interviews are recorded and analysed qualitatively, to get an in-depth understanding of management in hospitals. The record review method was reasonably well correlated with the IHMS score and offers an additional, related measure of management based on easily verifiable management items.

Our study had some limitations. First, we purposefully selected five interviewees per hospital to keep the data collection feasible, which meant we did not have the opportunity to gather insights from other hospital specialists. Furthermore, we prioritised some management domains over others in order to keep the tool at a length that was feasible to administer. For example, community or stakeholder engagement—a domain often assessed in other LMIC settings [10, 33]—could have been measured to evaluate whether hospitals consider community feedback and share performance data with the populations they serve. Similarly, the financial management domain could have been expanded to assess financial oversight by internal or external stakeholders [34].

The IHMS tool was developed for use in Malawian government and faith-based hospitals, with a focus on measuring management practices in neonatal units. There is potential for its integration into national policy [35] such as a standardised monitoring and assessment tool. For national-level implementation, the Government could consider periodic administration by central or regional assessors as part of quality improvement programmes. The frequency of administration would depend on available resources but given that management practices typically require several months to change meaningfully, annual administration could serve as a practical starting point. This approach could inform resource allocation decisions and strengthen accountability mechanisms.

At the operational level, the IHMS could support hospital managers to conduct internal evaluations or reviews with nearby peer hospitals, identifying areas for improvement and developing management implementation plans. Integration with local quality improvement activities could involve using IHMS results to guide hospital-specific management interventions, inform performance discussions, and track management practice improvements over time.

Our results suggested that the IHMS includes some practices currently outside hospital control (e.g. staff promotion) and others within hospital control but rarely implemented (e.g. structured handovers). Future implementers should consider whether to adapt the tool to measure only management practices that are already being implemented and within hospital control, or to also include practices that are either currently outside hospital control but could potentially be decentralised in future, and/or practices that are within hospital control but not currently being implemented. This decision hinges on whether implementers want to highlight the gap between current practice and evidence-based standards, or prioritise assessing only practices that are feasible within existing operational constraints. If they choose the former, IHMS results could help advocate for further decentralization or other policy changes.

The tool’s adaptability extends beyond neonatal care settings. The first domain, “Delivery of care in the neonatal unit,” can be modified for other hospital departments such as labour wards, paediatric units, or other medical wards. For those assessing overall hospital management rather than ward-specific practices, the first domain could be redesigned as ward-agnostic, with interviews conducted among respondents with hospital-wide knowledge. Those interested in understanding community engagement could consider adding a domain that focuses on community outreach to understand local concerns and share facility performance information, as has been implemented by others in the field [10, 33].

The IHMS could be adapted for hospitals in other low-resource settings, with modifications to reflect local governance structures (including hospital leadership, quality improvement teams, and drug committees), clinical protocols, and relevant institutional policies such as human resources policies. Management practices would need to be calibrated to reflect context-specific benchmarks to ensure meaningful interpretation.

Researchers can link IHMS results with quality of care outcome data for patients admitted to surveyed hospitals’ neonatal units, generating cross-sectional, observational evidence on potential associations between hospital management and quality of care. Previous researchers have employed this approach using both WMS and non-WMS tools [29]. Our research group applies this approach in a separate publication, linking IHMS results with data on clinical quality of care and in-hospital neonatal mortality for small and sick newborns admitted to the neonatal units of surveyed hospitals.

In conclusion, our findings indicate that the tool is feasible to implement, well-accepted by respondents, and demonstrates strong psychometric performance. We provide suggestions for how the IHMS could be integrated into national policy in Malawi as a research or monitoring tool, as well as recommendations for adaptation in other low-resource settings. This paper contributes to the limited evidence base on the development and performance testing of tools for quantitatively measuring management practices in the healthcare sector.

## Supplementary Information

Below is the link to the electronic supplementary material.

Supplementary Material 1

Supplementary Material 2

## Data Availability

Data can be requested from the corresponding or last author upon reasonable request.
