# Supplementary material for: Development and testing of the IMPRESS hospital management survey (IHMS) to measure hospital management practices in a low-income country setting
Source: BMC Health Serv Res. 2025 Nov 20;25:1496. doi: 10.1186/s12913-025-13704-7 (PMC12632003; doi:10.1186/s12913-025-13704-7)
Supplement: Supplementary file 1 — Supplementary Material 1 [file 12913_2025_13704_MOESM1_ESM.docx]

## Supplementary File

### Appendix 1. Management practices in published tools, listed by management domain

Operations

1. Lean operations (1)
2. Admitting the patient
3. Standardisation and protocols within unit
4. Coordination on handoffs
5. Communication among staff
6. Patient focus
7. Discharging the patient
8. Operations (2)
9. Layout of patient flow
10. Rationale for introducing standardisation/pathway management
11. Standardisation and protocols
12. Good use of human resources
13. Operations and monitoring (3)
14. Layout of patient flow
15. Rationale for introducing standardisation/pathway management
16. Continuous improvement
17. Performance tracking
18. Performance review
19. Performance dialogue
20. Operations (4)
21. Triage
22. Inventory of drug stock
23. Operations (5)
24. Operating hours
25. User fees displayed
26. Healthcare workers present 24 hours a day
27. Formal training
28. Handwashing station (soap and water)
29. Time spent on managerial activities
30. Care of patients (6)
31. Clinical support - operating theater services (6)
32. Clinical support - medication management (6)
33. Patient flow (7)
34. Pharmacy services (7)
35. Laboratory services (7)
36. Infection prevention safety (7)
37. Medical equipment management (7)
38. Operation (8)
39. Layout of patient flow
40. Rationale for improving patient management
41. Standardisation and protocols
42. Continuous improvement
43. Use of human resources
44. Operations management (9)
45. Medical environment
46. Layout of services
47. Patient-centred services
48. Clinical pathway
49. Continuous quality improvement
50. Lean process management (10)
51. Goals set internally
52. Goals publicised and widely supported
53. Standardised protocols in use and consistent with hospital goals
54. Essential surgical supplies and equipment obtained through a reliable, competitive procurement process
55. Staffing reflects hospital needs and employee skills
56. Operations management (11)
57. Layout of patient flow
58. Rational for introducing standardisation and pathway management
59. Standardisation and protocols
60. Good use of human resources
61. Target management (11)
62. Target balance
63. Target interconnection
64. Time horizon of the targets
65. Clearly defined accountability of clinicians
66. Clarity and comparability of targets
67. People management (11)
68. Rewarding high performers
69. Removing poor performers
70. Promoting high performers
71. Managing talent
72. Retaining talent
73. Attracting talent
74. Planned case scheduling (12)
75. Flexible physical capacity (12)
76. Bottlenecks (12)
77. Standardisation of process (12)
78. Labor floor efficiency (12)
79. Commitment to vaginal delivery (12)

Targets

1. Targets (1)
2. Target balance
3. Target interconnection
4. Target stretch
5. Targets (2)
6. Target balance
7. Target interconnection
8. Time horizon
9. Target stretch
10. Clarity and comparability of targets
11. Targets (3)
12. Target balance
13. Target interconnection
14. Time horizon
15. Target stretch
16. Clarity and comparability of targets
17. Targets (4)
18. Set targets
19. Targets (5)
20. Annual budget
21. Facility accountable to fixed population
22. Measure coverage of key population indicators
23. Planning and target setting (13)
24. Business plan update
25. Business plan content
26. Staff attention to business plan
27. Target update
28. Setting stretch/achievable targets
29. Staff attention to targets
30. Target (8)
31. Target balance
32. Target interconnection
33. Time horizon of the targets
34. Target stretch
35. Clearly defined accountability for clinicians
36. Clarity and comparability of targets
37. Target management (9)
38. Target balance
39. Target setting
40. Target sharing
41. Target stretch
42. Target management (11)
43. Target balance
44. Target interconnection
45. Time horizon of the targets
46. Clearly defined accountability of clinicians
47. Clarity and comparability of targets

Human resources

1. Incentives (1)
2. Rewarding high performers
3. Removing poor performers
4. Managing talent
5. Retaining talent
6. Human resources/people (2)
   1. Rewarding high performers
   2. Addressing poor performers
   3. Promoting high performers
   4. Managing talent
   5. Retaining talent
   6. Attracting talent
7. Incentives (3)
   1. Consequence management
   2. Rewarding high performers
   3. Addressing poor performers
   4. Promoting high performers
   5. Managing talent
   6. Retaining talent
   7. Attracting talent
8. Human resources (4)
   1. Training plan
   2. Formal system of staff appraisal
9. Human resources (5)
   1. Criteria for evaluating staff performance
   2. Supervisors review staff performance
   3. Offer training to staff
   4. Main method of supervision
10. Human resources (6)
11. Staff management (13)
    1. Staff involvement in bonus decisions
    2. Team work building
    3. Efforts to improve staff working environment
    4. Feedback to the officer in charge (OIC)
    5. Responses from OICs to feedback
    6. Open communication
    7. PBF bonus allocation
    8. Rewarding of high-performing staff
    9. Addressing low-performing staff
12. Human resource management (7)
13. Talent (8)
    1. Rewarding high performers
    2. Removing poor performers
    3. Promoting high performers
    4. Instilling a talent mindset/managing talent
    5. Retaining talent
    6. Attracting talent
14. Talent management (9)
    1. Rewarding
    2. Staff satisfaction
    3. Attracting talent
15. Sanctions for staff (14)
16. Talent management (10)
    1. Employees actively recruited
    2. Promotions and rewards based upon performance
    3. Underperforming employees reassigned or terminated
17. People management (11)
    1. Rewarding high performers
    2. Removing poor performers
    3. Promoting high performers
    4. Managing talent
    5. Retaining talent
    6. Attracting talent
18. Dynamic resource management (12)
19. Flexible nurse staffing (12)
20. Patient assignment (12)
21. Obstetrician availability (12)
22. Team collaboration (12)
23. Conflict management (12)
24. Human resources (15)
    1. Human resource management (HRM) staff
    2. HRM budget
    3. HR planning
    4. Job classification system
    5. Compensation and benefits system
    6. Recruitment, hiring, transfer, promotion
    7. Orientation Program for New staff
    8. Policy manual
    9. Discipline, Termination, and Grievance Procedures
    10. HIV/AIDS Workplace Prevention Program
    11. Employee data
    12. Computerisation of data
    13. Personnel files
    14. Staff retention strategy
    15. Job descriptions
    16. Staff supervision
    17. Work planning and performance review
    18. Staff in training
    19. Management and leadership development for staff
    20. Links to external preservice training

Performance/monitoring

1. Performance management (1)
   1. Technology adoption
   2. Monitoring errors/safety
   3. Continuous improvement
   4. Performance review
   5. Performance dialogue
2. Monitoring (2)
   1. Continuous improvement
   2. Performance tracking
   3. Performance review
   4. Performance dialogue
   5. Consequence management
3. Data/performance review (4)
   1. Use of patient records
   2. Use of the government health information system
   3. Monitoring patient indicators of quality
   4. Review of patient indicators
   5. Monitor business indicators
   6. Review of business indicators
4. Monitoring (5)
   1. Reporting new outbreaks
   2. Tracking common conditions
   3. Sharing and display of health data
   4. Reporting of client opinions
   5. Facility has quality improvement activities
   6. Discussing routine statistics with staff
   7. Books for tracking revenue and expenditure
   8. Data for service delivery are highly valued
5. Management of information (6)
6. Performance management (13)
   1. Visualisation of performance data
   2. Staff attention to performance
   3. Regular performance review meetings
   4. Performance review discussions
7. Performance quality improvement (7)
8. Medical records management (7)
9. Performance (8)
   1. Performance tracking
   2. Performance review
   3. Performance dialogue
   4. Consequence management
10. Performance management (16)
    1. Hospital performance appraisal
    2. Department performance appraisal
    3. Staff performance appraisal
    4. Penalties on staff with dissatisfactory performance
    5. Uneven workload
11. Performance-based funding for facility (14)
12. External supervision from a higher-level facility (14)
13. Monitoring (10)
    1. Adherence to standardised protocols monitored
    2. Progress toward quality improvement goals tracked and reviewed
    3. Surgical and executive leadership held accountable for progress toward goals
    4. Adverse events reported and reviewed (e.g., departmental meetings)
14. Performance monitoring (11)
    1. Continuous improvement
    2. Performance tracking
    3. Performance review
    4. Performance dialogue
    5. Consequence management

Financial management

1. Financial management (4)
   1. Annual budget
   2. Annual statement of revenue and expenditure
2. Use of funds and financial management (13)
   1. Drug management
   2. Use of PBF funds to attract patients and build trust
   3. Financial record update
   4. Financial record content
3. Financial management (7)
4. Financial oversight (10)
   1. Hospital finances examined by external entity
   2. Surgical and executive leadership responsible for financial well-being

Community/Stakeholder Engagement

1. Community engagement (5)
   1. Community advisory board meetings
   2. Made changes as a result of client opinion
   3. Collection of client opinions
   4. Community members regularly in staff meetings
   5. Shared performance of facility with community
   6. Patient opinions driving change and improvement
2. Patient rights and access to care (6)
3. Community/client engagement (13)
   1. Outreach
   2. Household visit
   3. Listening and responding to client feedback
   4. Patient recruitment and retention activities
4. Stakeholder engagement (13)
   1. Meetings with community leaders
   2. Request to community leaders
   3. Activities to encourage support from the community
   4. Meetings with supervisors
   5. Request to supervisors
5. Community participation via the community advisory council (14)

Leadership

1. Leadership and governance (7)
2. Leadership (2)
   1. Clearly defined accountability for clinicians

Autonomy/decentralised decision-making

1. Autonomy/decentralised decision-making (2)
   1. Who, in-hospital, decides to hire full-time permanent nurses
   2. Who, in-hospital, decides on more speciality beds
   3. Autonomy of departments in setting their own budgets and making investments
2. National governance for budgetary and expenditure decisions (14)
3. Municipal governance for budgetary and expenditure decisions (14)

## Appendix 2

Table 4 IHMS management items assessed in the record review by interviewers

| **No.** | **Whether the following management items were present:** |
| --- | --- |
|  | **Delivery of care in the neonatal unit** |
| 1 | Care of the Infant and Newborn in Malawi manual |
| 2 | Infection prevention and control manual |
| 3 | Poster displayed on infection prevention and control in neonatal unit (such as handwashing or waste disposal) |
| 4 | Neonatal referral forms |
| 5 | Neonatal death review form |
| 6 | Neonatal death audit consolidation form |
| 7 | Emergency box for neonatal care |
| 8 | Handover report or book in neonatal unit |
| 9 | Routine preventive maintenance schedules for neonatal equipment |
|  | **HR records** |
| 10 | Staff appraisal record in the personnel file |
| 11 | Continuous professional development schedule for hospital staff |
| 12 | Nurse roster in neonatal unit for month ahead |
| 13 | Clinician rota in neonatal unit for month ahead |
|  | **Quality/safety** |
| 14 | Targets for quality indicators in neonatal unit |
| 15 | Performance data on quality of care indicators in neonatal unit |
| 16 | Display of information on ombudsman’s office in neonatal unit |
| 17 | Suggestion box in neonatal unit |
| 18 | Targets for hospital |
|  | **Finance** |
| 19 | Hospital budget for 2022/23, available in hospital |
| 20 | Hospital expenditure against each budget line, available in the hospital |
|  | **Leadership and governance** |
| 21 | Minutes/records of hospital senior management meeting |
| 22 | Minutes/records of Quality Improvement Support Team meetings |
| 23 | Minutes/records of infection prevention and control meetings |
| 24 | Minutes/records of Work Improvement Teams meetings in neonatal unit |
| 25 | Display of posters on Work Improvement Teams activities in neonatal unit |

Table 5 Mean scores for individual management practices and interitem correlations

| **Item** | **Mean** | **Std. dev** | **Average interitem correlation** | **Cronbach’s Alpha** |
| --- | --- | --- | --- | --- |
| 1. Layout | 4.15 | 0.76 | 0.311 | 0.924 |
| 2. Triage | 3.54 | 0.70 | 0.300 | 0.920 |
| 3. COIN protocols | 3.83 | 0.61 | 0.295 | 0.919 |
| 4. IPC protocols | 3.81 | 0.55 | 0.299 | 0.920 |
| 5. Handover | 3.40 | 0.57 | 0.310 | 0.924 |
| 6. Referrals | 3.25 | 0.85 | 0.300 | 0.921 |
| 7. Audit | 3.46 | 0.82 | 0.300 | 0.920 |
| 8.Supervision | 2.88 | 1.03 | 0.298 | 0.920 |
| 9. Equipment | 4.03 | 0.61 | 0.292 | 0.918 |
| 10. Appraisal | 3.08 | 1.04 | 0.293 | 0.918 |
| 11. Promotion | 2.23 | 0.64 | 0.297 | 0.920 |
| 12. Reward | 2.56 | 1.02 | 0.303 | 0.921 |
| 13. Poor performance | 3.56 | 0.51 | 0.300 | 0.921 |
| 14. Recruitment | 2.79 | 0.52 | 0.292 | 0.918 |
| 15. Temporary staff | 3.64 | 0.78 | 0.292 | 0.918 |
| 16. Staff allocation | 4.01 | 0.55 | 0.291 | 0.917 |
| 17. Capacity strengthening | 3.58 | 0.72 | 0.294 | 0.918 |
| 18. Monitoring errors | 2.40 | 0.79 | 0.298 | 0.920 |
| 19. Performance review | 2.53 | 0.82 | 0.292 | 0.917 |
| 20. User satisfaction | 3.41 | 0.67 | 0.295 | 0.919 |
| 21. Target range | 3.06 | 0.74 | 0.293 | 0.918 |
| 22. Target communication | 2.96 | 0.77 | 0.289 | 0.917 |
| 23. Budget setting | 3.70 | 0.66 | 0.306 | 0.923 |
| 24. Budget expenditure | 3.56 | 0.57 | 0.304 | 0.922 |
| 25. Senior leadership governance | 3.25 | 0.93 | 0.302 | 0.921 |
| 26. Quality of care governance | 3.85 | 0.59 | 0.293 | 0.918 |
| 27. Drug procurement | 3.81 | 0.62 | 0.298 | 0.920 |
| 28. IPC governance | 3.42 | 0.78 | 0.309 | 0.924 |
| **Overall** |  |  | **0.299** | **0.922** |
| COIN: Care of the Infant and Newborn in Malawi  IPC: Infection Prevention and Control | | | | |

### References

1. McConnell KJ, Lindrooth RC, Wholey DR, Maddox TM, Bloom N. Management Practices and the Quality of Care in Cardiac Units. JAMA Internal Medicine. 2013;173(8):684-92.

2. Tsai TC, Jha AK, Gawande AA, Huckman RS, Bloom N, Sadun R. Hospital board and management practices are strongly related to hospital performance on clinical quality metrics. Health Aff (Millwood). 2015;34(8):1304-11.

3. Bloom N, Propper C, Seiler S, Van Reenen J. The Impact of Competition on Management Quality: Evidence from Public Hospitals. The Review of Economic Studies. 2015;82(2):457-89.

4. Powell-Jackson T, King JJC, Makungu C, Quaife M, Goodman C. Management Practices and Quality of Care: Evidence from the Private Health Care Sector in Tanzania. Econ J (London). 2024;134(657):436-56.

5. Macarayan EK, Ratcliffe HL, Otupiri E, Hirschhorn LR, Miller K, Lipsitz SR, et al. Facility management associated with improved primary health care outcomes in Ghana. PLOS ONE. 2019;14(7):e0218662.

6. Dunsch FA, Evans DK, Eze-Ajoku E, Macis M. Management, supervision, and healthcare: A field experiment. Journal of Economics & Management Strategy. 2023;32(3):583-606.

7. Fetene N, Canavan ME, Megentta A, Linnander E, Tan AX, Nadew K, et al. District-level health management and health system performance. PLOS ONE. 2019;14(2):e0210624.

8. Yoo SGK, Davies D, Mohanan PP, Baldridge AS, Charles PM, Schumacher M, et al. Hospital-Level Cardiovascular Management Practices in Kerala, India. Circulation: Cardiovascular Quality and Outcomes. 2019;12(5):e005251.

9. Zhu Y, Zhao Y, Dou L, Guo R, Gu X, Gao R, et al. The hospital management practices in Chinese county hospitals and its association with quality of care, efficiency and finance. BMC Health Services Research. 2021;21(1):449.

10. Funk LM, Conley DM, Berry WR, Gawande AA. Hospital management practices and availability of surgery in sub-Saharan Africa: a pilot study of three hospitals. World J Surg. 2013;37(11):2520-8.

11. Agarwal R, Green R, Agarwal N, Randhawa K. Management practices in Australian healthcare: can NSW public hospitals do better? J Health Organ Manag. 2016;30(3):331-53.

12. Plough AC, Galvin G, Li Z, Lipsitz SR, Alidina S, Henrich NJ, et al. Relationship Between Labor and Delivery Unit Management Practices and Maternal Outcomes. Obstet Gynecol. 2017;130(2):358-65.

13. Mabuchi S, Alonge O, Tsugawa Y, Bennett S. Measuring management practices in primary health care facilities – development and validation of management practices scorecard in Nigeria. Global Health Action. 2020;13(1):1763078.

14. Salas-Ortiz A, La Hera-Fuentes G, Nance N, Sosa-Rubí SG, Bautista-Arredondo S. The relationship between management practices and the efficiency and quality of voluntary medical male circumcision services in four African countries. PLoS One. 2019;14(10):e0222180.

15. Management Sciences for Health. Human Resource Management Rapid Assesment Tool For Health Organizations Cambridge, MA2012 [Available from: <https://msh.org/wp-content/uploads/2021/12/hrm_tool_version_3_2012_final_webv.pdf>.

16. Zhu Y, Guo R, Dou L, Zhao Y, Li S, Qiao Y, et al. Development and evaluation of a hospital management practice rating scale. Journal of Hospital Administration. 2018;7:9.
