# Supplementary material for: Development and testing of the IMPRESS hospital management survey (IHMS) to measure hospital management practices in a low-income country setting
Source: BMC Health Serv Res. 2025 Nov 20;25:1496. doi: 10.1186/s12913-025-13704-7 (PMC12632003; doi:10.1186/s12913-025-13704-7)
Supplement: Supplementary file 2 — Supplementary Material 2 [file 12913_2025_13704_MOESM2_ESM.docx]

| **IMPRESS Hospital Survey of Management Practices** |
| --- |

Section 1: Basic characteristics

| Interview Information | Manager Information |
| --- | --- |
| Date: _____________________________  [DD/MM/YYYY]  Start time: ______________________________  [24 HOUR CLOCK]  Hospital ID: ______________________________  [USE CODING SHEET]  District: _____________________________  [NAME OF DISTRICT]  Type of hospital: ________________________________  [GOVERNMENT/CHAM/CENTRAL]  Hospital name: _________________________________  [NAME OF HOSPITAL]  Name of interviewer: __________________________________  [DROP DOWN]  Name of note taker: ________________________________________ [DROP DOWN] | Category of manager being interviewed __________________________________________________________________________  [SISTER IN-CHARGE OF NNU; UNIT MATRON; ADMINISTRATOR; SENIOR NURSE MANAGER; SENIOR MEDICAL MANAGER]  What is your current job title: _____________________________________________________________________________________________  [CURRENT JOB TITLE]  *[Unit matron only]* When did you start this in this position at this hospital? _______________________________________________  [MONTH/YEAR]  *[Unit matron only]* When did your predecessor start in the position ___________________________________________________  [MONTH/YEAR; DON’T KNOW]  What is your highest education qualification? _____________________________________________________________________  DIPLOMA; DEGREE; MASTERS; PhD; OTHER (SPECIFY)  Do you have a management related qualification? ________________________  [YES/NO]  [If yes]: What is the management qualification? ___________________________________________________________  [BSc HEALTH MANAGEMENT; MBA HEALTH MANAGEMENT; OTHER (SPECIFY)  (*Unit matron/sister-in-charge only)* Are you trained on COIN guidelines? ___________________________________________  [YES; NO]  *(Unit matron/sister-in-charge only)* Are you trained on antimicrobial stewardship? Is it within IPC training?__________________________  [YES, WITHIN IPC TRAINING; YES, SEPARATE FROM IPC TRAINING; NO]  How long have you worked in this hospital? ____________________________________________________________  CATEGORIES <1; 1 to 2; 3 to 5; 6 to 9; 10 or more YEARS  How long have you worked in this district? __________________________________________________________________  CATEGORIES <1; 1 to 2; 3 to 5; 6 to 9; 10 or more YEARS  Gender of participant: __________________________________________________________  [MALE; FEMALE]  What is your age? ____________________________________________________________________  [YEARS] |

Section 2: Interview

| 1. **DELIVERY OF CLINICAL CARE IN THE NEONATAL UNIT** | | | | | | | | |
| --- | --- | --- | --- | --- | --- | --- | --- | --- |
| 1. **Layout to optimise patient flow for the neonatal unit**   ***Tests how well the layout is configured to optimise patient flow*** | | | | | | | | |
| - 1. Is your neonatal unit close to the labour ward? | | | Yes, next to □  Yes, close to □  No □ | | | | | |
| - 1. Can you briefly describe the layout of the neonatal unit?   2. What is the thinking or rationale behind this layout?   3. To what extent does the layout help infection prevention and control? Can you give some examples of how it helps? | | | 1□ 2□ 3□ 4□ 5□ | | | | | |
| Score 1: Layout of neonatal unit does not optimise patient flow. Neonatal unit and labour ward are not close and there is no clear rationale for the layout. | | Score 3: Layout of neonatal unit has been thought-through and optimized as far as possible. | Score 5: Neonatal unit layout has been designed to optimize patient flow and promote infection prevention and control; neonatal unit is next/close to the labour ward | | | | | |
| 1. **Triage for newborns**   ***Tests if hospital has a functioning triage system to identify, assess and provide appropriate care for newborns with life-threatening problems*** | | | | | | | | |
| 1. Does the neonatal unit have a triage system to sort newborns into different risk groups? | | | Yes □  No □ | | | | | |
| 1. Tell me about the triage system for newborns. How familiar are staff with the standardised triage guidelines, such as those in COIN? 2. Is there a newborn emergency box? Is it fully equipped and ready to use at all times? 3. How often do you organise emergency drills for the triage of newborns? | | | 1□ 2□ 3□ 4□ 5□ | | | | | |
| Score 1: No triage system exists in the newborn unit. | | Score 3: Triage system exists but is not fully standardised or used consistently. | Score 5: Triage system according to standardised triage guidelines is known and used consistently. Emergency box is always available and emergency drills are done. | | | | | |
| 1. **Protocols for management of illness for small and sick newborns**   ***Tests if there are standardised protocols for small and sick newborns that are applied and monitored systematically*** | | | | | | | | |
| 1. Are all staff familiar with the COIN protocols for small and sick newborns? | | | | | | Yes, all staff □  Some staff □  No staff □ | | |
| 1. To what extent are COIN protocols followed for different conditions? 2. What tools and checklists do health workers use? Can you give a few examples (e.g. scoring gestational age; assessing pain)? 3. Are health workers monitored to ensure they are following COIN protocols? How is this done? | | | | | | 1□ 2□ 3□ 4□ 5□ | | |
| Score 1: COIN protocols are not known or used by health workers in the neonatal unit; procedures are not standardised. | | Score 3: COIN protocols exist in the neonatal unit but are not commonly used. Health workers’ use of COIN protocols are not commonly monitored. | Score 5: COIN protocols are known and used by all health workers consistently and regularly followed up on through monitoring or oversight | | | | | |
| 1. **Standardisation of protocols for infection prevention and control in the neonatal unit**   ***Tests if there are standardised procedures for infection prevention and control that are applied and monitored systematically*** | | | | | | | | |
| - 1. Are all health workers familiar with the infection prevention and control protocols in the neonatal unit? | | | | | | Yes, all staff □  Some staff □  No staff □ | | |
| - 1. To what extent are infection prevention and control processes followed for different IPC practices in the neonatal unit (hand hygiene, decontamination of devices and equipment, environmental cleaning, outbreak detection, aseptic techniques)?   2. How do supervisors monitor whether health workers are following the established clinical protocols for IPC? Are any tools or checklists used? | | | | | | 1□ 2□ 3□ 4□ 5□ | | |
| Score 1: IPC protocols are not known or used by health workers in the neonatal unit; IPC procedures are not standardised. | | Score 3: IPC protocols exist in the neonatal unit but are not commonly used and not monitored adequately. | Score 5: IPC protocols are known and used by all health workers and regularly followed up on through some form of monitoring or oversight | | | | | |
| 1. **Handover between shifts**   ***Tests whether the hospital has a standardised process for health workers’ handover between shifts*** | | | | | | | | |
| - 1. Do you have a standardised process for health workers’ shift handover? If yes, how often do staff comply with the standardised process? | | | | | | Yes, all of the time □  Yes, most of the time □  Yes, some of the time □  No system used □ | | |
| - 1. Tell us about how handovers are done? Is this done for both clinicians and nurses?   2. Do nurses regularly use written notes for handover?   3. Do clinicians regularly use written notes for handover?   4. How are managers able to ensure that a standardised process for handovers is followed? | | | | | | 1□ 2□ 3□ 4□ 5□ | | |
| Score 1: There are no systems in place for health workers to pass information between each other between their shifts. | | Score 3: A standardised process exist but is not used all the time or by all clinicians and nurses. There is some monitoring of compliance. | Score 5: A standardised process exists for handover and is used by clinicians and nurses. The process is regularly monitored for compliance. | | | | | |
| 1. **System for receiving referrals from other health facilities**   ***Tests whether the hospital has a standardised process for receiving neonatal referrals*** | | | | | | | | |
| 1. Are there protocols in place for managing the referrals of neonates to this facility? | | | | | | Yes □ No □ | | |
| 1. Can you tell me about the referral system for receiving neonatal patients? What protocols are in place? To what extent is hospital transport available for referrals? 2. How does the hospital communicate with the referring facility? 3. Is feedback provided to the referring facility after receiving the patient? | | | | | | 1□ 2□ 3□ 4□ 5□ | | |
| Score 1: There is no system in place to standardise inward referral of patients; there is little communication between the referring facility and the hospital. There is no organised transport. | | Score 3: Some referral standardisation exists; a referral communication system exists but may not be adhered to. Feedback between facilities is ad hoc. | Score 5: A standardised process for receiving inward referrals exists. Referral communication is functional using a referral form and organised transport. There is feedback between facilities. | | | | | |
| 1. **Audit of neonatal deaths**   ***Tests whether audit is used as an effective tool for improving clinical practices*** | | | | | | | | |
| 1. Do you audit neonatal deaths in this facility? How frequently do you do this? | | | | | | Yes, all of the time □  Yes, most of the time □  Yes, some of the time □  No system used □ | | |
| 1. How does the system for auditing neonatal deaths work? Is there a feedback process with action points and follow up? 2. Do you complete neonatal death review forms? To what extent are they completed within 72 hours? 3. Are neonatal death audit review meetings happening regularly? When was the last one? | | | | | | 1□ 2□ 3□ 4□ 5□ | | |
| Score 1: There is no system in place to standardise auditing of neonatal deaths. | | Score 3: Some standardisation for auditing neonatal deaths exists; audit forms exist but may not be used frequently and the process is not monitored closely or adhered to rigorously. | Score 5: A standardised process for auditing neonatal deaths exists; standard audit forms for these deaths are completed within 72 hours; there are regular mortality audit review meetings with actionable outcomes and follow up. | | | | | |
| 1. **Supervision**   ***Tests whether the hospital has a system in place for supervising health workers in the neonatal unit*** | | | | | | | | |
| - 1. Does supervision of health workers in the neonatal unit by hospital managers happen? | | | | | | | | Every week □  Every month □  Every quarter □  Less than every quarter □  Never □ |
| - 1. How is supervision conducted in the neonatal unit? Is it supportive and constructive?   2. Does the process involve demonstration of how to do things correctly?   3. Are standard tools available to support supervision? | | | | | | | | 1□ 2□ 3□ 4□ 5□ |
| Score 1: Supervision is unstructured, not documented and does not follow a regular schedule. Tools to support supervision are not available or used. Supervision is not supportive. | | Score 3: Some structure and tools exist for supervision. Supervision is conducted infrequently. | Score 5: Supervision is conducted weekly, using standardised tools. Supervision is supportive and involves demonstration. | | | | | |
| 1. **Equipment management in the neonatal unit**   ***Tests whether the hospital has a system in place for preventive maintenance and repair of equipment in the neonatal unit*** | | | | | | | | |
| - 1. Do you have a well-functioning system for preventive maintenance in the neonatal unit? How about for repair of equipment? | | | Yes, preventive and repair □  Yes, repair only □  Yes, preventive only □  No □ | | | | | |
| - 1. Do you have a system for logging malfunctioning of equipment, communicating with technicians and recording actions taken? How well does it work?   2. What system is there in place for routinely servicing equipment in the neonatal ward? How frequently is this conducted?   3. To what extent are maintenance staff trained and skilled to fix equipment in the neonatal unit? When equipment is broken, how quickly does it get fixed? | | | 1□ 2□ 3□ 4□ 5□ | | | | | |
| Score 1: There is no system in place to communicate with staff to fix equipment. Equipment is often broken and preventive maintenance does not happen. | Score 3: The system to communicate with staff to fix equipment is not fully functional and staff to fix equipment are not always trained. There are delays to fixing equipment. | | Score 5: There is a system in place to communicate with trained staff to fix equipment in a timely manner. Preventive maintenance is carried out routinely. | | | | | |
| 1. **HUMAN RESOURCE MANAGEMENT FOR HEALTH WORKERS** | | | | | | | | |
| 1. **Appraisal system**   ***Tests whether the hospital has a formal system to appraise the performance of healthcare workers*** | | | | | | | | |
| 1. Do you have an appraisal system for health workers? | | | | | | Yes □ No □ | | |
| 1. How does your appraisal system work? Do you have criteria / guidelines for appraising staff? Can you give an example? 2. To what extent do the appraisals happen as frequently as they are meant to? Are there any consequences for non-completion of the appraisal? 3. Do you use the appraisal results to improve performance and development of health workers? 4. Is it done for all cadres of health worker? | | | | | | 1□ 2□ 3□ 4□ 5□ | | |
| Score 1: There is no system in place to appraise the performance of healthcare workers. | | Score 3: Some healthcare workers complete and submit the appraisal but it is not universal; appraisals are not done annually; the process is not standardised and not monitored closely or adhered to rigorously. | Score 5: The majority of health workers complete and submit the appraisal at least once a year. The system specifies a formal set of criteria to evaluate performance. Completion of appraisals is monitored and there are consequences for not completing the appraisal. Appraisal results are used to improve performance and capacity. | | | | | |
| 1. **Promoting high performing health workers**   ***Tests whether promotion of health workers is based primarily on job performance*** | | | | | | | | |
| 1. Does the hospital have any influence on promotion decisions for health workers? | | | Substantial influence □  Some influence □  No influence □ | | | | | |
| 1. To what extent do you feel that better performing health workers generally get promoted faster? 2. How are better performers identified? 3. Are there any other factors influencing promotion decisions? | | | 1□ 2□ 3□ 4□ 5□ | | | | | |
| Score 1: People are promoted primarily on the basis of tenure. Hospital has limited influence on promotion decisions for its health workers. | | Score 3: Promotions are somewhat influenced by performance, alongside other factors such as tenure. Promotion decisions involve hospital managers but to a limited degree. | Score 5: Promotions are strongly influenced by performance. Hospital managers have influence on promotion decisions. Irrelevant factors (e.g. nepotism or politics) do not play a role. | | | | | |
| 1. **Rewarding high performing health workers**   ***Tests whether good individual performance is rewarded (financial or otherwise) proportionately*** | | | | | | | | |
| 1. Does the hospital have any system of rewarding or recognizing well performing health workers? | | | | | | Yes □ No □ | | |
| 1. What are the different ways health workers are rewarded or recognised for good performance? Are there non-financial rewards for good performance? Can you explain how this system works? 2. Are rewards based on well-defined criteria? 3. Are rewards available for all cadres of health worker? | | | | | | 1□ 2□ 3□ 4□ 5□ | | |
| Score 1: Health workers are not rewarded or are rewarded irrespective of performance level | | Score 3: There is a system in place that rewards or recognises individuals but it is for some cadres only and is based on ad hoc or poorly defined performance measures | Score 5: There is a system which rewards or recognises individuals from all cadres based on performance; rewards are awarded as a consequence of well-defined and monitored individual achievements | | | | | |
| 1. **Dealing with poorly performing health workers**   ***Tests whether hospital can deal with underperformers (including use of staff sanctions)*** | | | | | | | | |
| 1. Does the hospital have clear disciplinary procedures in the event of severe poor performance or misconduct? [If yes] is it possible in practice to dismiss people? | | | | | | No system not functional □  Yes system functional, cannot dismiss staff □  Yes system functional, can dismiss staff □ | | |
| 1. If you had a health worker with severe poor performance or misconduct, what would happen? Could you give me a recent example? 2. How complicated is the disciplinary process? How long does it take? 3. Is it possible to move poor performers to less critical roles? 4. Do some poorly performing individuals rarely face being disciplined? | | | | | | 1□ 2□ 3□ 4□ 5□ | | |
| Score 1: Poor performers are rarely removed from their positions | | Score 3: It takes several years for poor performers to be removed from their positions. Only the most severe forms of misconduct are acted upon. | Score 5: There are clear disciplinary procedures which are followed in a timely manner. It is possible to move poor performers to less critical roles and if necessary to dismiss staff. | | | | | |
| 1. **Recruiting skilled health workers on a permanent basis**   ***Tests whether hospital has the ability to identify and recruit skilled health workers on a permanent basis*** | | | | | | | | |
| 1. Does the hospital have any influence on the recruitment of health workers on a permanent basis? | | | | | | Substantial influence □  Some influence □  No influence □ | | |
| 1. How do you forecast recruitment needs for the neonatal unit? 2. Tell me about the process for recruiting a new nurse on a permanent basis. What about for a clinician? 3. How long does it typically take to recruit a health worker, say a nurse? 4. To what extent do you feel that those who get recruited are the best candidates? Are there any other factors influencing hiring decisions? | | | | | | 1□ 2□ 3□ 4□ 5□ | | |
| Score 1: There is no system of forecasting recruitment needs. Recruitment of health workers is very slow. Those that are hired are rarely the most competent – irrelevant factors appear to play a major role in hiring decisions. | | Score 3: There is a system of forecasting recruitment needs but the information is not always acted upon. The process for recruiting skilled health workers to permanent contracts is not timely but does tend to identify the more competent candidates. | Score 5: Systematic process for forecasting gaps, identifying and recruiting skilled health workers to permanent contracts in a timely manner. Irrelevant factors (e.g. nepotism or politics) do not play a role. | | | | | |
| 1. **Hiring temporary and locum health workers**   ***Tests whether hospital can forecast and address gaps in critical staff through temporary and locum workers*** | | | | | | | | |
| 1. Do you have a well-functioning system for hiring temporary and locum nurses to address staff shortages? | | | | | Yes □ No □ | | | |
| 1. How do you identify the need for temporary or locum staff for nurses and clinicians? 2. What is the process for hiring temporary and locum nurses? What about clinicians? How well do these processes work? 3. Is it possible to recruit temporary and locum staff with the skills needed for working in the neonatal unit? | | | | | | 1□ 2□ 3□ 4□ 5□ | | |
| Score 1: There is no system for forecasting temporary and locum staffing needs. The process for hiring temporary and locum nurses does not function such that it is rarely done by the hospital. | | Score 3: The hospital has a system for forecasting and addressing temporary and locum staffing needs but gaps are not always filled or those hired are not always appropriately skilled. | Score 5: Well-functioning system in place to forecast and address critical staff gaps and to hire appropriately skilled locum and temporary staff to fill these. | | | | | |
| 1. **Allocation of health workers to the neonatal unit**   ***Tests whether hospital allocates health workers to roles they are best qualified for*** | | | | | | | | |
| 1. Is there a system of allocating health workers to different departments based on department needs and health worker skills? | | | | | | | Yes □ No □ | |
| 1. Tell me about the process for allocating health workers to the neonatal unit? What influences the allocation? 2. Is there regular communication between the neonatal unit and hospital management on staffing allocation? | | | | | | 1 2 3 4 5 | | |
| Score 1: The allocation of health workers across units, including the neonatal unit, takes no account of the skills and experience of staff. | | Score 3: A process exists for assessing which departments need what skills but it is not always applied systematically. Staff with skills in neonatal care tend to be allocated to the neonatal unit but not always. | Score 5: Hospital has a responsive and systematic approach to assessing which staff are needed by the neonatal unit and allocating staff accordingly. There is two-way communication and feedback between the hospital and neonatal unit to discuss staffing allocation. | | | | | |
| 1. **Programme for capacity strengthening**   ***Tests whether hospital has a programme for capacity strengthening to improve skills of health workers*** | | | | | | | | |
| 1. Does the hospital have a training plan based on a systematic assessment of needs? | | | | Yes □ No □ | | | | |
| 1. How does the hospital assess the capacity strengthening needs of its health workers? 2. How is it decided what training sessions are held? 3. Tell me about the CPD sessions? How often are they? How well are they attended? | | | | 1 2 3 4 5 | | | | |
| Score 1: Hospital does not have a programme in place for capacity strengthening. | | Score 3: Hospital has a programme in place for capacity strengthening activities but it is not tailored to the needs of health workers and health workers attend on an ad hoc basis. | Score 5: Hospital has a programme in place to plan capacity strengthening activities for staff on a regular basis according to the needs of the health workers. Sessions are well attended. | | | | | |
| 1. **HOSPITAL AND NEONATAL WARD LEVEL TARGET SETTING AND MONITORING OF PERFORMANCE** | | | | | | | | |
| 1. **Monitoring medical errors or harmful practices**   ***Tests whether hospital has systems in place for detecting harmful practices*** | | | | | | | | |
| - 1. Do you have a system where medical errors or harmful practices (e.g. medication errors, wrong procedure) are reported? If yes, is it used? | | | | | | | Yes system, yes used □  Yes, system, not used □  No □ | |
| 1. Can you tell me about your systems for avoiding harmful practices? What are the measures in place? For example, do you use an incident report form? 2. How would you know if an individual was not following a safety protocol in the neonatal unit? 3. Has the hospital ever managed to make improvements after detecting a medical error? What happened? | | | | | | | 1□ 2□ 3□ 4□ 5□ | |
| Score 1: There is little awareness of the importance of avoiding harmful practices. There is no system for reporting medical errors or harmful practices. Safety depends on individual efforts only. | | Score 3: Systems for reporting medical errors or harmful practices do exist but are rarely used. Medical errors are addressed primarily through broader quality improvement efforts (e.g. QIST, death audits). | Score 5: Systems for avoiding/reducing harmful practices are in place and monitored, for example, supervisors regularly investigate medical errors and this leads to changes to reduce potential harm to patients. | | | | | |
| 1. **Performance review**   ***Tests whether hospital managers monitor hospital performance of patient and quality of care indicators in the hospital*** | | | | | | | | |
| - 1. How often does the DHMT / senior management formally review hospital quality of care indicators? | | | | | | | Monthly  Every 3 months □  Twice a year □  Once a year □  Never □ | |
| - 1. What type of indicators are reviewed? Do any measure clinical quality of care? What are the sources of information? Can you give an example?   2. Tell me about the review meetings of hospital performance. Do they happen at the neonatal ward level?   3. Is a review report made? Who gets to see it?   4. What is a typical follow-up plan that results from these reviews? | | | | | | | 1□ 2□ 3□ 4□ 5□ | |
| Score 1: Performance is reviewed infrequently and focuses primarily on patient volume indicators. Formal reports are rarely produced. | | Score 3: Performance is reviewed every quarter and includes some quality of care indicators. Review reports are produced but they are not shared widely and no clear follow-up plans are adopted. | Score 5: Performance (patient and quality of care indicators) is reviewed monthly in management meetings, review reports are made and are available to managers, and all aspects are followed up to ensure continuous improvement | | | | | |
| 1. **User satisfaction**   ***Tests whether the hospital uses patient or family feedback and uses evidence for improvement*** | | | | | | | | |
| 1. Is there a system that routinely captures patient or family feedback on their experience of care? | | | | | | | Yes □ No □ | |
| 1. Tell me about any systems in place to capture patient or family questions or concerns about their care? Do these systems cover the neonatal ward? If no, what happens in the neonatal ward? 2. What processes exist to escalate concerns that are not immediately resolved by health workers? 3. Have you made any changes based on the feedback from patients? Can you give an example? | | | | | | | 1□ 2□ 3□ 4□ 5□ | |
| Score 1: Patient satisfaction is rarely measured; no systems are in place to capture patient or family questions or concerns. | | Score 3: Systems to measure patient satisfaction and capture patient feedback exist but are not comprehensive. Efforts tend to be sporadic. There are no protocols to respond to patient feedback. | Score 5: Multiple systems are functioning to capture patient/family concerns (e.g. exit interviews, suggestions box, hospital ombudsman); protocols are in place to respond to feedback. | | | | | |
| 1. **Setting an appropriate range of targets**   ***Tests whether the targets for the hospital and neonatal unit cover a sufficiently broad set of metrics*** | | | | | | | | |
| - 1. Does the hospital have specific numerical targets for different indicators? | | | | | | | Yes □ No □ | |
| 1. What types of targets are set for the hospital? Which areas do they cover? Are there targets for the neonatal unit? Can you give an example? 2. How are the targets set? Who is involved in target setting? 3. How tough are your targets to achieve - are you pushed by them? Do you ever achieve these targets? | | | | | | | 1□ 2□ 3□ 4□ 5□ | |
| Score 1: There are no hospital specific targets covering clinical care. | | Score 3: There are hospital specific targets covering different areas of clinical care. They may be set by the central level in the first instance and, where appropriate, revised by the hospital with some consultation. The targets set are sometimes far too easy or too difficult to achieve. | Score 5: Targets cover key areas of clinical care, including the neonatal ward. Targets are tailored to the hospital and are set through a consultative process involving managers and health workers across the hospital. Hospital is pushed by the targets. | | | | | |
| 1. **Clarity and communication of targets**   ***Tests whether targets are easily understandable and openly communicated*** | | | | | | | | |
| - 1. Are these targets communicated to staff at all levels? | | | | | | | Yes, at all levels □  Yes, at some levels  No □ | |
| 1. How easy or difficult are the targets for the hospital staff to understand? Is this true of the neonatal ward? 2. How are targets communicated to staff? Are these targets displayed to staff? How are they displayed? 3. To what extent are targets known and understood by different levels of staff? | | | | | | | 1□ 2□ 3□ 4□ 5□ | |
| Score 1: There are no hospital specific targets or the targets that exist are complex and not easily understood; there is no awareness of the targets beyond the senior management. | | Score 3: Targets are well defined and communicated to managers across the hospital but health workers have little awareness of them and there no display of the targets around the hospital | Score 5: Targets are well-defined, clearly communicated and well understood by staff at all levels. Targets are displayed around the hospital and reinforced at all levels. | | | | | |
| 1. **FINANCIAL MANAGEMENT** | | | | | | | | |
| 1. **Budget setting**   ***Tests whether the hospital has consultative and systematic process for setting the annual budget*** | | | | | | | | |
| 1. Do you have an annual budget preparation meeting that involves all key stakeholders? | | | Yes, all stakeholders □  Yes, most stakeholders □  Yes, some stakeholders □  No □ | | | | | |
| 1. How do you assess and quantify the needs of the hospital when proposing your annual budget? 2. In the previous financial year, did your approved annual budget match with the annual budget you proposed? If not, how did you overcome this? 3. In the previous financial year, did the approved monthly budget match with the actual monthly budget you were finally allocated? If not, how did you overcome this? | | | 1□ 2□ 3□ 4□ 5□ | | | | | |
| Score 1: Hospital budget is prepared with little or no consultation outside of senior management. It is based largely on previous year’s budget with no assessment of the evolving needs of the hospital. Submitted budget bears little relation to the approved budget. | | Score 3: Budget preparation involves hospital departments but the process to prioritise these competing needs is not consultative or transparent. Differences between the proposed, approved and actual budget are managed by a small group of senior managers. | Score 5: Budget preparation involves key stakeholders (hospital departments, district council, civil society). There is a systematic process for prioritising the needs of the hospital when proposing the annual budget and for managing differences between the proposed, approved and actual budget. | | | | | |
| 1. **Reviewing expenditure against the budget**   ***Tests whether hospital has an up-to-date statement of hospital revenue and expenditure*** | | | | | | | | |
| 1. Do you have a well-functioning system for regularly comparing expenditure against the budget? | | | | Yes, well-functioning □  Yes, somewhat functioning □  No □ | | | | |
| 1. How is the financial position of the hospital monitored? By financial position we mean what is in your budget, how much money is at hand, what debts need to be settled and the projections for the remaining at hand. Who is involved? 2. How frequently does this happen? 3. How are the results of these reviews used and communicated to the budget users? Can you adjust within the budget? 4. Is there a systematic process for coping with delays? 5. If the budgets did not match, what measures did you take to address the deficit? | | | | 1□ 2□ 3□ 4□ 5□ | | | | |
| Score 1: There is no awareness of the financial position of the hospital beyond the accountant. There is no systematic process for dealing with delays. | | Score 3: Financial position is reviewed quarterly but the expenditure report is typically not made available for the DHMT. Delays and deficits are sometimes managed through a systematic process. | Score 5: Financial position is reviewed in detail every month by the accountant, in liaison with DHMT and council and results are regularly communicated to all budget users. There is a strategy for dealing with delays and deficits. | | | | | |
| 1. **LEADERSHIP AND GOVERNANCE** | | | | | | | | |
| 1. **Senior leadership governance**   ***Tests whether hospital has a functional hospital management team*** | | | | | | | | |
| 1. Does the hospital senior management team have a terms of reference (TOR) and an implementation plan? | | | Yes, both □  Yes, TOR only  Yes, plan only  No □ | | | | | |
| 1. Tell me how the hospital management team functions? How frequently do they meet? 2. Who is represented on the hospital management team? 3. How is the performance of the hospital management team monitored in achieving targets in their implementation plan? 4. How does the hospital management team communicate with other levels of staff in the hospital? | | | 1□ 2□ 3□ 4□ 5□ | | | | | |
| Score 1: The hospital management team is represented by a small subset of senior leaders, does not have clearly defined roles and responsibilities or implementation plan. They meet and communicate with hospital staff rarely | | Score 3: The hospital management team is somewhat functional but there are major gaps in the way they define, approve and monitor the implementation plan. Communication with hospital staff is infrequent. They meet infrequently. | Score 5: A multidisciplinary hospital management team meets regularly; they regularly define, approve and monitor the implementation plan and regularly communicate with hospital staff. | | | | | |
| 1. **Quality of care governance**   ***Tests whether hospital has a functional quality improvement support team (QIST) and neonatal ward level work improvement team (WIT)*** | | | | | | | | |
| 1. Does the hospital have QIST? Does the neonatal ward have a WIT? | | | Yes, both □  Yes, QIST only  Yes, WIT only  No □ | | | | | |
| 1. How does the QIST and WIT function? Do they have TORs? How frequently do they meet? 2. Who is represented in the QIST? Who is represented in the WIT? 3. How are the activities of the QIST monitored? 4. How are the activities of the WIT monitored? | | | 1□ 2□ 3□ 4□ 5□ | | | | | |
| Score 1: Hospital level QIST and neonatal ward level WIT are non-existent or not functional. | | Score 3: Hospital level QIST and neonatal ward level WIT are somewhat functional but there are major gaps in the way they define roles and responsibilities and monitor activities. They meet infrequently. | Score 5: Hospital level QIST and neonatal ward level WIT exist and are represented by key stakeholders; they meet regularly; Activities are monitored. | | | | | |
| 1. **Procurement process for medicines and supplies for the neonatal unit**   ***Tests the functionality of the procurement systems to get medicines and supplies for the neonatal unit*** | | | | | | | | |
| 1. Does the hospital have a functioning drug and therapeutic committee? | | | Yes, □  No, □ | | | | | |
| 1. How does the drug and therapeutic committee operate? 2. Is there a system for accurately forecasting needs for medicines and supplies for the neonatal unit? 3. What systems do you have to communicate between the neonatal unit and the hospital pharmacy? 4. How in practice do you address shortages of medicines and supplies in the neonatal unit? | | | 1□ 2□ 3□ 4□ 5□ | | | | | |
| Score 1: The drug and therapeutics committee does not function. The neonatal unit does not forecast needs for medicines and there are no strategies for addressing shortages. | | Score 3: The drug and therapeutics committee is somewhat functional, needs for the neonatal unit are not always forecasted accurately or communicated. There some strategies for addressing shortages of medicines and supplies. | Score 5: There is a functional drug and therapeutics committee. Needs for the neonatal unit are accurately forecasted, well communicated and there are strategies for addressing shortages of medicines and supplies. | | | | | |
| 1. **Governance for infection prevention control**   ***Tests whether hospital has a functional IPC programme*** | | | | | | | | |
| - 1. Does an infection prevention and control committee exist in the hospital? | | | Yes □ No □ | | | | | |
| - 1. How does the committee function? Are minutes taken? Are follow-up steps documented?   2. Who is on the committee? Is it multi-disciplinary?   3. Is there a specific item on the IPC committee meeting agenda for antimicrobial stewardship? | | | 1□ 2□ 3□ 4□ 5□ | | | | | |
| Score 1: Infection prevention and control committee does not exist or it does not function at all. | | Score 3: Infection prevention and control committee exists but it does not meet regularly; the purpose and follow-up steps of these meetings are not always clear and minutes are not always taken. | Score 5: Multi-disciplinary infection prevention and control committee exists and meets regularly; meetings have a purpose and minutes are taken; follow-up steps are documented. Antimicrobial stewardship is often included in the agenda | | | | | |

Section 3: Post-interview

This section will be completed after the interview by both research assistants to reflect on how the interviewee responded to the questions.

| - 1. Interviewee knowledge of management practices | | 1 2 3 4 5 |
| --- | --- | --- |
| Score 1: Some limited knowledge about his/her area of work, and no knowledge about the rest of the hospital | Score 3: Expert knowledge about his/her area of work, and some limited knowledge about the rest of the hospital | Score 5: Expert knowledge about his/her specialty and the rest of the hospital |
| 1. Interviewee willingness to reveal information | | 1 2 3 4 5 |
| Score 1: Very reluctant to provide more than basic information | Score 3: Provides all basic information and some more confidential information | Score 5: Totally willing to provide any information about the hospital! |
| 1. Interviewee patience | | 1 2 3 4 5 |
| Score 1: Little patience - wants to run the interview as quickly as possible. I felt heavy time pressure | Score 3: Some patience - willing to provide richness to answers but also time constrained. I felt moderate time pressure | Score 5: Lot of patience - willing to talk for as long as required. I felt no time pressure |

Section 4: Hospital record review

In this section, the interviewer will ask to see evidence of some management practices or processes for confirmation.

| **No.** | **Question** | **Response (score)** |
| --- | --- | --- |
|  | **Delivery of care in the neonatal unit** |  |
| 1 | COIN manual | Yes (1) No (0) |
| 2 | IPC (infection prevention and control) manual | Yes (1) No (0) |
| 3 | Poster displayed on IPC in neonatal unit (such as handwashing or waste disposal) | Yes (1) No (0) |
| 4 | Neonatal referral forms *(ask to see a blank form)* | Yes (1) No (0) |
| 5 | Neonatal death review form *(ask to see a blank form)* | Yes (1) No (0) |
| 6 | Neonatal death audit consolidation form *(ask to see most recently completed form)* | Yes, with a date DD/MM/YY (2) Yes, without a date (1) No (0) |
| 7 | Emergency box for neonatal care | Yes (1) No (0) |
| 8 | Handover report or book in neonatal unit | Yes (1) No (0) |
| 9 | Routine preventive maintenance schedules for neonatal equipment | Yes (1) No (0) |
| 10 | Number of neonatal admissions in the most recent completed calendar month | [Number]  [Specify the month of the most recent completed month] |
|  | **HR records** |  |
| 11 | Staff appraisal record in the personnel file *(at least one from either 2021 or 2022 is acceptable)* | Yes (1) No (0) |
| 12 | CPD (continuous professional development) schedule for hospital staff *(schedule should be forward looking for 2022/2023)* | Yes (1) No (0) |
| 13 | Nurse roster in NNU for month ahead | Yes (1) No (0) |
| 14 | Clinician rota in NNU for month ahead | Yes (1) No (0) |
|  | **Quality / safety** |  |
| 15 | Targets for quality indicators in NNU | Yes displayed (2) Yes not displayed (1) No (0) |
| 16 | Performance data on quality of care indicators in NNU *(performance data for either 2021 or 2022 are acceptable)* | Yes displayed (2) Yes not displayed (1) No (0) |
| 17 | Display of information on ombudsman’s office in NNU | Yes (1) No (0) |
| 18 | Suggestion box in NNU | Yes (1) No (0) |
| 19 | Targets for hospital | Yes displayed (1) Yes not displayed (2) No (0) |
|  | **Finance** |  |
| 20 | Hospital budget for 2022/23, available in hospital *(either paper or electronic is acceptable)* | Yes (1) No (0) |
| 21 | Hospital expenditure against each budget line, available in the hospital *(Ask to see the latest report and explain that you don’t want to review the actual details)* | Updated as of last month (1)  Updated as of three months ago (2)  Updated as of six months ago (3)  Not available (0) |
|  | **Leadership and governance** |  |
| 22 | Minutes / records of hospital senior management meeting | Yes – Date DD/MM/YY (2) Yes – Not dated (1) No (0) |
| 23 | Minutes / records of QIST meetings | Yes – Date DD/MM/YY (2) Yes – Not dated (1) No (0) |
| 24 | Minutes / records of IPC meetings | Yes – Date DD/MM/YY (2) Yes – Not dated (1) No (0) |
| 25 | Minutes / records of Work Improvement Teams (WITS) meetings in NNU | Yes – Date DD/MM/YY (2) Yes – Not dated (1) No (0) |
| 26 | Display of posters on WITS activities in NNU | Yes (1) No (0) |
| 27 | What is the gender of the current head of the hospital? | Male; Female; Don’t know |
| 28 | Do you know when the current head of the hospital started in their role?  *(Hospital Director or Director of Health and Social Services, Medical Superintendent. If the respondent only knows the month and year, put the first day of the month.* | Yes; No  [Select date] |
| 29 | What is the gender of the current head of the hospital? | Male; Female; Don’t know |

Please take GPS coordinates of the facility
